# FOXM1 targeting alters AURKB activity and reshapes antitumor immunity to curb the progression of small cell lung cancer

**DOI:** 10.21203/rs.3.rs-6960266/v1

**Published:** 2025-07-01

**Authors:** Md Arafat Khan, Parvez Khan, Mahek Fatima, Asad Ur Rehman, Laiba Anwar, Zahraa Wajih Alsafwani, Aatiya Ahmad, Mohammad Ali Abbas Zaidi, Jesse L. Cox, Areem Zahid, Sameer Mohiuddin, Sung Hoon Kim, Juan A Santamaria-Barria, Imayavaramban Lakshmanan, Benita S Katzenellenbogen, John A. Katzenellenbogen, Apar K Ganti, Surinder K Batra, Mohd Wasim Nasser

**Affiliations:** University of Nebraska Medical Center; University of Nebraska Medical Center; University of Nebraska Medical Center; University of Nebraska Medical Center; University of Nebraska Medical Center; University of Nebraska Medical Center; University of Nebraska Medical Center; University of Nebraska Medical Center; University of Nebraska Medical Center; University of Nebraska Medical Center; University of Nebraska Medical Center; University of Illinois Urbana-Champaign; University of Nebraska Medical Center; University of Nebraska Medical Center; University of Illinois Urbana-Champaign; University of Illinois Urbana-Champaign; University of Nebraska Medical Center; University of Nebraska Medical Center; University of Nebraska Medical Center

**Keywords:** Small cell lung cancer, FOXM1 inhibitors, T cell activation, chemotherapeutic resistance, metastasis

## Abstract

**Background:**

Small cell lung cancer (SCLC) is a lethal lung malignancy and patients are often diagnosed with distant metastasis. Nearly all patients suffer from disease relapsing with inherent chemoresistance. Lack of targeted SCLC therapies further worsens disease outcomes, making it highly desirable to identify novel and effective therapeutic targets.

**Methods:**

To search for potential therapeutic targets in SCLC, we analyzed publicly available single-cell and bulk RNA-sequencing (RNA-seq) data from normal, lung adenocarcinoma, and SCLC tumor tissues. To assess the targeting potential of FOXM1, we developed various *in vitro* models, including DOX-On-shFOXM1 (Tet-ON) inducible stable knockdown systems. Cisplatin resistant human and murine SCLC cell lines were generated to assess the role of FOXM1 in chemotherapy resistance. Immunoblotting, immunohistochemistry (IHC), and immuno-fluorescence were used to analyze the expression of FOXM1 and target proteins. ChIP-assay was used to study protein-gene interactions. Further, multicolor flow cytometry was employed to study the effect of FOXM1 inhibition on human T cells activation and differentiation. Subcutaneous xenograft and SCLC spontaneous (RPM: *RB*^*fl/fl*^*;TP53*^*fl/fl*^*;LSL-MYC*^*T58A*^) mouse models were used to evaluate the efficacy of FOXM1 inhibitors.

**Results:**

Single-cell as well as bulk RNA-seq data revealed that FOXM1, an oncogenic transcription factor, is overexpressed in SCLC, and it was recapitulated in human and murine SCLC tissues and cell lines. Interestingly, chemo-resistant (CR) SCLC showed a substantially higher FOXM1 expression than naïve SCLC. Silencing FOXM1 genetically or pharmacologically by FOXM1 inhibitors revealed a marked reduction in cell viability, colony formation, migration and sphere formation in naïve and CR SCLC cells. Moreover, FOXM1 inhibition induced apoptosis and cell cycle arrest in SCLC cells. Furthermore, FOXM1 inhibition in combination with first-line platinum-based chemotherapy showed synergistic anticancer effects in both xenograft and RPM mouse models of SCLC. Our RNA-seq analysis revealed that FOXM1 inhibition altered the Aurora Kinase B (AURKB) signaling pathway, which is dysregulated in SCLC. Moreover, we found FOXM1 inhibition enhanced T cell activation and supported the differentiation of CD8 + cytotoxic T cells, and T cell-mediated killing of cancer cells.

**Conclusions:**

Our study demonstrates that FOXM1 targeting using small molecule inhibitors has the potential to be a novel therapeutic strategy to combat SCLC progression including chemotherapeutic resistance and reshaping the anti-tumor immune response.

## Background

Small cell lung cancer (SCLC) remains an aggressive and recalcitrant malignancy. Platinum-based chemotherapy and etoposide effectively regress SCLC and have been used as the first-line therapy for SCLC patients [1]. Eventually, SCLC patients show relapse, accompanied by chemoresistance (CR), and succumb to the disease due to limited therapeutic efficacy [2]. Although immunotherapeutic drugs like PD-L1 inhibitors have been approved for SCLC as first-line therapy, only a small number of SCLC patients benefit from immunotherapy combined with chemotherapy [3]. This is mostly due to the immune evasive mechanisms employed by the SCLC cells [4, 5]. A lack of targeted therapy has long plagued SCLC patients. However, a gap in knowledge of SCLC biology has been the biggest impediment for the development of novel therapeutic strategies. A targeted therapy in combination with conventional chemotherapy has the potential to increase overall and progression-free survival in most SCLC patients.

Recent progress in SCLC research helped to understand the involvement of different transcription factors (TFs), including ASCL1, NEUROD1, POU2F3, MYC, and YAP1 in categorizing the various subtypes of SCLC [6, 7]. The TFs play a key role in SCLC heterogeneity and modulate disease progression; however, the targeting of these TFs is not successful as these modulators are also involved in physiological processes and no small molecule is available to target ASCL1, NEUROD1, or POU2F3. In addition to these subtype defining TFs, recently, our group and one more study has shown that Forkhead box M1 (FOXM1), a transcription factor that belongs to the Forkhead Box (FOX) transcription factor family, plays a key role in SCLC growth and metastasis [8, 9]. Indeed, FOXM1 has been identified as an indispensable component for SCLC progression and metastasis [8, 10]. Moreover, it was observed that high FOXM1 expression in SCLC patients correlated with advanced stage of the disease, extra-thoracic metastases, and poor overall survival (OS) compared to low FOXM1 group [9]. Our recent study established that the CXCR4-FOXM1-RRM2 axis played a substantial role in SCLC growth and metastases [8].

FOXM1 is essential for embryonic development or embryogenesis; however, its expression ceases in terminally differentiated cells [11, 12]. FOXM1 plays a significant role in cell cycle progression, cellular proliferation, cellular differentiation, intracellular metabolism, redox signaling, tissue homeostasis, apoptosis, angiogenesis, and DNA damage repair [13–15]. Consistent with FOXM1’s role in cell cycle progression and proliferation, FOXM1 also plays a crucial role in various malignancies [11, 15, 16]. FOXM1 overexpression has been observed in multiple human cancers, including breast adenocarcinoma [17, 18], ovarian cancer [19], prostate cancer [20], nasopharyngeal carcinoma [21], cervical cancer [22], head and neck squamous cell carcinoma [23] and many others. The genomic location of the FOXM1 gene is on chromosomal band 12p13.33 in humans and amplification of this segment is a common scenario in FOXM1-associated cancers [11, 24]. The recent studies indicate that FOXM1 played an essential role in various aspects of tumorigenesis or tumor progression, including cell growth, metastasis, metabolic adaptations, and modulation of tumor immune response [8, 9, 17, 25], suggesting that it is a therapeutic vulnerability that can be exploited to develop potential therapies for multiple tumor types, including SCLC.

Since FOXM1 is one of the major oncogenic TF, efforts to develop FOXM1 inhibitors have been initiated and as a result, a plethora of compounds with FOXM1 inhibitory activities have been developed/identified. Naturally occurring compounds like curcumin, honokiol, extract of solanum incanum, genistein and diarylheptanoids have been found to either decrease FOXM1 expression and its downstream targets or weaken the gene network of FOXM1 [11, 26]. Two thiazole antibiotics including Siomycin A and thiostrepton displayed their ability to inhibit FOXM1 in transformed lung fibroblast and breast cancer cell line models [27, 28]. Afterward, proteasome inhibitors bortezomib and carfilzomib were shown to suppress FOXM1 [29]. However, these compounds lacked FOXM1 selectivity; restricting their use for FOXM1-targeted therapies and also showing many off-targeting effects [30]. During the present decade, efforts have been made to develop novel and selective inhibitors targeting FOXM1, including FDI-6, NB-73, NB-115, and RCM-1 [18, 31–33]. Among these different FOXM1 inhibitors the most well studied ones are FDI-6 and NB compounds, and they have been extensively studied in breast cancer and to some extent in ovarian cancer and multiple myelomas [15, 19, 34]. The NB compounds are based on a 1,1-diarylethylene core and multiple modifications have been made to this core, which led to the development of several analogues [39]. Interestingly, RCM-1 blocks the interaction between FOXM1 and β-catenin, leading to the inhibition of both molecules [32]. On the other hand, STL-001 is another novel class of FOXM1 inhibitor which can potently relocalize nuclear FOXM1 to the cytoplasm where it promotes its autophagic degradation [42]. FDI-6 binds to the DNA binding domain of FOXM1 and hampers subsequent transactivation by FOXM1 [11, 18, 31]. FDI-6 has been tested by multiple research groups and shown significant anticancer properties [17, 30, 35].

The outcomes of recent studies have attracted the interest of the scientific community to evaluate the efficacy of FOXM1 inhibitors in different tumor types [13, 17–19, 25]. Aforementioned above, our recent study along with others established the involvement of FOXM1 in SCLC growth and metastasis, revealing a promising platform to evaluate FOXM1 targeted therapies in SCLC progression. In the present study, we evaluated the efficacies of FOXM1 selective small molecule inhibitors alone or in combination with first-line chemotherapy on SCLC progression, including chemotherapeutic resistance using SCLC xenografts and spontaneous models. The findings deepen our understanding that FOXM1 targeting in SCLC modulates AURKB and T cell mediated immune response, suggesting that combining FOXM1 inhibitors with chemo- and/or immunotherapy has the potential to improve clinical outcomes of this aggressive disease.

## Materials and Methods

### RNA-sequencing and differential gene expression analysis

DMS-273 and DMS-273 FDI-6 treated cells were treated with RNA lysis buffer provided by mirVana Kit from Thermo Fischer Scientific (Catalogue number- AM1561). Total RNA isolation was carried out according to the protocol provided by the manufacturer. To ensure the quality of RNA for sequencing, a test was performed using Agilent Bioanalyzer (by Agilent Technologies, Inc) to assess RNA quality. The RNA samples with an RNA integrity number (RIN) of 10 were considered eligible for sequencing. The entire process for the preparation of library, amplification using PCR, distribution of size, library quantification and subsequent sample loading were all carried out using established protocol [8]. Rapid mode RNA sequencing experiment was performed by our collaborators at City of Hope, CA, USA. A single end read utilizing 50 cycles was used for sequencing along with onboard clustering and V2 chemistry. The sequencing core facility generated raw RNA-sequencing data, and it was provided to us. HiSeq2500 sequencer (Illumina Inc, CA) manufactured by Illumina was used to perform the sequencing experiment using rapid mode. Raw files generated in this RNA-sequencing experiment are going to be submitted to NCBI SRA.

### SCLC xenograft mouse model study using luciferase tagged SBC3 cells

SBC3 cells were transduced using lentiviral particles containing firefly-luciferase constructs. Upon successful transduction, the transformed SBC3 cells were able to emit light by catalyzing D-Luciferin using the firefly-luciferase. This was tested in IVISbrite imager from PerkinElmer. After determining the bioluminescence of the SBC3-Luc cells, 2 million cells were subcutaneously implanted on the right flank of NSG mice aging 8 weeks and above. Male and female animals were used in this experiment in approximately equal ratio. Experimental animals were monitored every day for tumor initiation. Once the mice developed palpable tumors, they were subdivided into four distinct treatment groups- 1) vehicle control, 2) cisplatin monotherapy group, 3) FDI-6 monotherapy group and 4) combination treatment group (cisplatin and FDI-6) and treatment were initiated. Cisplatin and FDI-6 were injected intraperitoneally two times a week. IVIS bioluminescence imaging was carried out to follow the growth of tumors. Moreover, tumor volume was measured using a digital slide caliper. Standard equation for subcutaneous tumor volume was utilized: tumor volume (mm^3^) = (tumor width)^2^ × length/2. After 4 weeks of treatment the mice were euthanized and tumors along with mice organs were harvested and fixed in formaldehyde (37%). Later the tumors and organs were sent to the tissue sciences facility of University of Nebraska Medical Center for sectioning. The experiments and treatments performed under this experiment were in accordance with the requirements set by NIH (National Institute of Health) and IACUC (Institutional animal care and use committee).

### Spontaneous SCLC mouse model study in genetically engineered RPM mice

To further test the effectiveness of FDI-6 in animal models, an in vivo experiment was conducted on our spontaneous RPM mouse model. To generate SCLC spontaneously in our RPM mice, adenoviral mediated Cre recombinase was administered intranasally (10^7^ per mice). These recombinant adenoviruses mediated cre recombination reaction in RPM mice knocked out (KO) RB1 and P53 tumor suppressors and overexpressed Myc-luciferase. After KO of Rb1, P53 and Myc overexpression, the mice developed spontaneous tumors within a week or two. The progression of SCLC tumor was measured using IVIS bioluminescence imaging as the tumor cells expressed firefly luciferase which is present in the Myc cassette. Once the mice expressed a detectable bioluminescence signal, we subdivided the mice in four treatment groups- 1) vehicle control group, 2) cisplatin monotherapy group, 3) FDI-6 monotherapy group and 4) combination treatment group (cisplatin and FDI-6). The mice were treated for four weeks since the detection of bioluminescence signal. During this treatment period, tumor progression was monitored with IVIS bioluminescence imaging, twice a week. At the end of this treatment period, mice were euthanized, and mice organs were harvested. The organs were fixed and sent for tissue sectioning. The tissue sections were further taken used for H&E and IHC experiments. The mice treated under this experiment were raised and treated according to NIH and IACUC protocols.

## Results

### FOXM1 expression increases in SCLC cell lines and tissue samples

**Figure 1:**
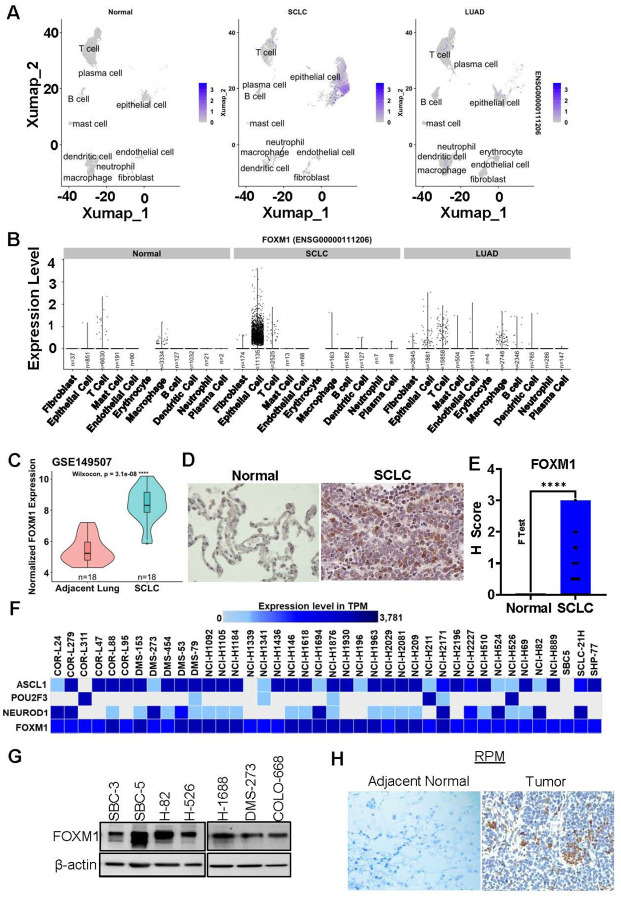
FOXM1 is overexpressed in small cell lung cancer samples. **A)** SCLC epithelial cell population is unique and different than normal and NSCLC epithelial cell population. **B)** FOXM1 is substantially overexpressed in SCLC epithelial cells only compared to normal or NSCLC cells. **C)** FOXM1 expression in SCLC data set (GSE149507)**. D)** Representative photographs of FOXM1 expression in SCLC tumor tissues. **E)** H scoring of FOXM1 expression in SCLC and normal lung tissues. **F)** Expression of FOXM1 in association with lineage specific markers (NE or Non-NE) in SCLC cell lines. **G)** FOXM1 expression in SCLC cell lines validated using western blotting. **H)** FOXM1 expression status in adjacent normal and tumor tissues in RPM lung. Statistical analysis is from student’s t test, unpaired comparison, **** = p <0.0001.

Recently, it has been shown by Liang et al. and our group that FOXM1 is among the top putative TFs modulating SCLC tumorigenesis and that it cooperates with metastasis-associated molecules such as CXCR4 [8, 9]. To further expand our understanding on the targeting potential of FOXM1 in SCLC, we interrogate the expression status of FOXM1 in different cell types using single-cell RNA-seq (scRNA-Seq) dataset of SCLC (HTAN MSK SCLC Single Cell Dataset, grant number- 1U2CCA233284–01) [36]. This scRNA-Seq dataset also contains the data from adjacent normal lung and lung adenocarcinoma (LUAD), which is the most prominent subtype of non-small cell lung cancer (NSCLC) Analyzing the single-cell dataset revealed different subpopulations of cells, including various types of immune cells (T cells, B cells, mast cells, dendritic cells, macrophages, and neutrophils), fibroblasts, endothelial, plasma, and epithelial cell types (Fig. 1A and supplementary Fig. 1A). It was observed that FOXM1 is overexpressed in SCLC cells compared to normal and LUAD cells (Fig. 1A–B and supplementary Fig. 1B). Further, the cell type specific analysis suggests that FOXM1 expression is associated with SCLC epithelial cell types, (Fig. 1A–B). The transcript level expression analysis showed that the FOXM1 expression is substantially high in SCLC epithelial cells compared to adjacent normal lung or LUAD epithelial cells, whereas low to null expression of FOXM1 was observed in other cell types (Fig. 1B). To further confirm, we analyzed another publicly available microarray dataset for SCLC patients (GSE149707), that showed significantly higher expression of FOXM1 mRNA relative to adjacent normal lung tissues (Fig. 1C and supplementary Fig. 1B). We further validated the expression of FOXM1 in human SCLC tissues microarray (Cat#BS04116a, US Biomax, Inc.) using immunohistochemical approach, and observed significantly higher expression of FOXM1 in SCLC tissues compared to normal lung tissues (Fig. 1D–E). In addition, we also analyzed FOXM1 level in all the publicly available SCLC cell line models in Expression Atlas. Interestingly, all SCLC cell lines showed high expression of FOXM1 irrespective of the subtype markers (Fig. 1F). We also studied FOXM1 protein expression in a panel of SCLC cell lines and observed high expression of FOXM1 in all of the human SCLC cell lines as shown (Fig. 1G).

Next, we wanted to validate our findings in vivo, and for this, a spontaneous mouse model of SCLC (RPM: *RB*^*fl/fl*^*;TP53*^*fl/fl*^*;LSL-MYC*^*T58A*^) was used, where we can perform conditional deletion of tumor suppressors RB1 and P53, and overexpression of MYC and it recapitulates human SCLC characteristics [37]. Upon *Adeno-CGRP-Cre* infection in the lungs, it resulted in the loss of RB1, P53 and MYC overexpression, which eventually develop murine SCLC tumors [38]. The SCLC tumors from RPM mice lung showed prominent expression of FOXM1 (Fig. 1H,). The outcomes of gene as well as protein level expression studies using scRNA-Seq, cell lines, and tumor tissues derived from human and spontaneous SCLC mouse model suggest that SCLC cells showed substantially higher expression of FOXM1 compared to normal lung and/or LUAD, indicating that FOXM1 is a key TF for SCLC that could be evaluated for targeted therapies.

### FOXM1 knockdown reduces colony formation, migration and spheroid growth of SCLC cells

**Figure 2:**
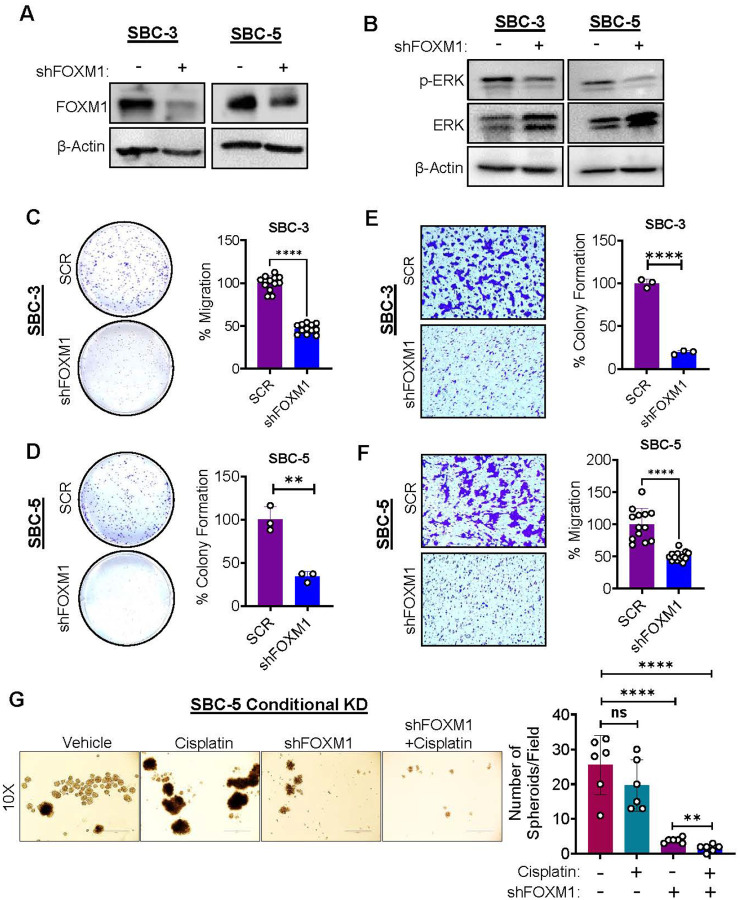
FOXM1 knockdown reduces FOXM1 protein expression, colony formation, migration and spheroid growth of SCLC cells. **A)** Conditional knockdown of FOXM1 decreases FOXM1 protein level. **B)** FOXM1 conditional KD decreases oncogenic ERK activation (/p-ERK). **C-D)** FOXM1 conditional KD significantly reduces colony formation capabilities of SCLC cells. **E-F)** FOXM1 KD significantly reduces migration of SCLC cells through the trans-well membrane. **G)** FOXM1 KD reduces the number of spheroids per field of vision compared to uninduced controls and the anti-tumor response is increased in combination with cisplatin. Statistical analysis is from student’s t test, unpaired comparison, ns=non-significant, **=p<0.001, and **** = p <0.0001.

To explore the potential role of FOXM1 in terms of targeted therapies, we first interrogated if FOXM1 is critical for SCLC growth as previous FOXM1-related studies in SCLC mainly focused on mouse model [9]. For this, we developed stable doxycycline inducible knock down (KD) models of FOXM1 in human SCLC cell lines using a Tet-on conditional KD system. In this KD system, the addition of doxycycline (doxycycline induction) allowed the expression of FOXM1 shRNA, which can bind to and degrade FOXM1 mRNA. We optimized the doxycycline dose through kill curves for inducing FOXM1 KD without affecting the growth of control cell lines. After doxycycline induction, protein expression studies were performed and a substantial reduction of FOXM1 protein was observed in SBC-3 and SBC-5 cell lines, which validated a successful KD of FOXM1 (Fig. 2A). After validating FOXM1 KD at protein levels, we immunoblotted for phospho-ERK (p-ERK) levels which is a crucial regulator of cell growth, invasion, and cancer metastasis [39]. It was observed that FOXM1 KD decreased the phosphorylation of ERK (Fig. 2B). Moreover, we performed colony formation assays to see the impact of FOXM1 KD on SCLC cell growth or proliferation. Interestingly, a significant reduction in the colony formation capabilities of FOXM1 KD cells (SBC3 and SBC5) were observed compared to their uninduced controls (Fig. 2C–D). As our ERK activation studies showed that FOXM1 KD inhibited the ERK activation (Fig. 1B), next we checked the effect of FOXM1 KD on the cell migration potential of SCLC cell lines. For this, a trans-well migration assay was performed after doxycycline induction and observed a significant reduction in the number of migrated cells in FOXM1 KD groups compared to their respective uninduced controls (Fig. 2E–F). Majority of SCLC cells show stem cell-like properties and can be grown easily in suspension or low attachment conditions [8]. Therefore, we performed a three-dimensional spheroid assay using conditional KD cells (SBC-5 conditional KD cells). Conditional KD of FOXM1 resulted in the reduction of total number of spheroids formed compared to uninduced/control or cisplatin treated cells, and the effects were augmented in combination treatment with cisplatin (Fig. 2G). Altogether, the data suggests that stable KD or depletion of FOXM1 protein using conditional KD system significantly attenuates SCLC colony formation, metastases and spheroid growth, indicating that FOXM1 is a good target for developing targeted therapies in SCLC.

### Targeting FOXM1 using small molecule inhibitors decreases viability of naïve and chemoresistant SCLC cells

**Figure 3:**
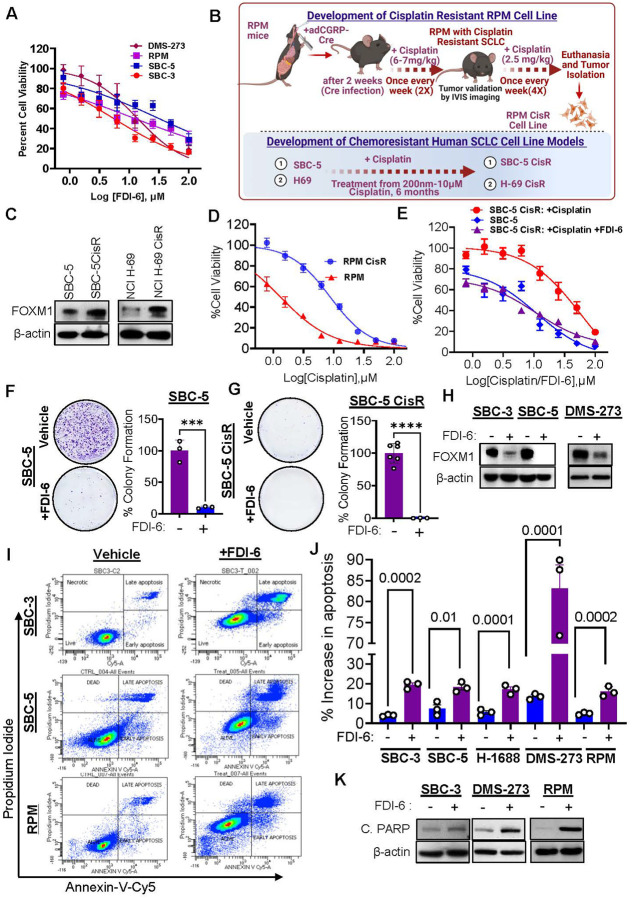
FOXM1 inhibitor (FOXM1i) treatment reduces viability, colony formation and induces apoptosis in SCLC cell lines. **A)** FDI-6 treatment reduces viability of human and murine SCLC cell lines. **B)** FOXM1 expression status in Cisplatin resistant SCLC cell lines compared to their naïve counterparts. **C)** Graphical representation of the strategy to generate cisplatin resistant SCLC cell lines. **D)** Comparison between naïve and cisplatin resistant RPM cell lines response towards cisplatin treatment. **E)** FOXM1 inhibition sensitizes cisplatin resistant SBC-5 CisR cells towards cisplatin. **F-G)** FOXM1 inhibition decreases colony formation of naïve and chemo-resistant SCLC cell lines (student’s t test, unpaired comparison, *** = p <0.0008, **** = p< 0.0001). **H)** FOXM1 inhibition using FDI-6 (5 μM for 48 h) reduces FOXM1 protein levels. **I)** FACs analysis reveals induction of apoptosis after FOXM1 inhibition in SCLC cells. **J)** Quantitation of apoptosis. **K)** Treatment of FDI-6 induces cleaved Caspase 3 (C. Caspase 3) expression.

Based on the FOXM1 expression and KD data that suggested FOXM1 as a potential therapeutic target, we evaluated the efficacy of selective FOXM1 inhibitors (FOXM1i) available, including FDI-6 and NB-73, for FOXM1 targeting in SCLC [31, 33]. Multiple cell lines of human SCLC (SBC-3, SBC-5, DMS-273, and H69) along with syngeneic mice cell line RPM were utilized for accessing the efficacies of FOXM1i using cell viability assays. Substantial reduction in cell viability was observed in all the cell line models with IC_50_ values ranging from 0.2–2 μM (Fig. 3A, Supplementary Fig. S2A-B). Since, SCLC patients universally encountered the relapse with acquire resistance to chemotherapy, we were interested to see, if FOXM1 inhibition can be used to target these resistant cells or sensitize them to cisplatin. Therefore, to understand the involvement of FOXM1 in CR, we generated cisplatin resistant (CisR) cells for RPM mouse model as well as human SCLC cell lines (Fig. 3B, C) that were validated using cell viability assays that revealed a 10–15-fold boost in the IC_50_ values for cisplatin in CisR cells compared to their respective naïve counterparts (Fig. 3D–E)). Further we checked the status of FOXM1 in CisR cells, and we found significant upregulation of FOXM1 in CisR cell lines (Fig. 3B). FOXM1 inhibitors were also tested on human and murine CisR-SCLC cell lines (Fig. 3E, Supplementary S2E-G). The cell viability studies showed that FOXM1i (FDI-6, NB-73) effectively decreased the viability of CisR-SCLC cell lines (Fig. 3E, supplementary S2E-G).

### FOXM1 inhibition sensitizes naïve and chemo-resistant SCLC cells to first-line chemotherapy with synergistic effects

Combining FOXM1 inhibitors with platinum based chemotherapeutic agents (cisplatin or carboplatin) might have a more drastic effect in aggressive SCLC cells as FOXM1 is known to confer chemoresistance in some other cancers [40]. To validate our hypothesis, we performed combination cell viability assays with FDI-6 and cisplatin. Interestingly, a fixed dose of FDI-6 selected from dose-response curve (2 μM for SBC-3, and 3 μM for DMS-273) in combination with cisplatin resulted in a synergistic antitumor response in multiple cell lines of human SCLC (supplementary Fig. S2C-D). As a result, low concentration of FDI-6 treatment in combination with cisplatin showed a more marked reduction of cell viability that was unseen in any single agent treatment. Moreover, FDI-6 treatment in combination with cisplatin sensitized the CisR-SCLC cells (SBC-5 CisR and RPM CisR, and H69 CisR) cells towards cisplatin, showing synergistic effects (Fig. 3E, supplementary Fig. S2H-I). These cells previously did not respond well to cisplatin and showed IC_50_ values several folds higher than the naïve cells (supplementary Fig. S2E). Interestingly, combining FDI-6 or NB-73 with cisplatin pushes the effectiveness of cisplatin activity profile on CisR-SCLC cell lines similar to their naïve counterparts (supplementary Fig. S2E,F). On top of that, a fixed dose of cisplatin in combination with a variable dose of FDI-6 did not show noticeable synergistic effect on cell viability (supplementary Fig. S2J). This result pointed at an insidious role of FOXM1 in the development of SCLC drug resistance. CR is a universal problem for SCLC patients, and the limited number of second line therapies has shown treatment benefits in these patients with an advanced disease. This is a remarkable advantage brought up by the utilization of FOXM1i in SCLC that can be exploited in both naive and CR resistant disease conditions. Altogether, the data suggests that FOXM1 inhibition sensitizes CR-SCLC cells to first line chemotherapy.

### FOXM1 inhibitors reduce colony formation, migration and spheroid growth of naïve and CR-SCLC cells

To evaluate the efficacies of FOXM1i in SCLC growth, we performed colony formation assays on naïve and CR-SCLC cell lines. For this, human and murine SCLC cell lines were treated with FOXM1 inhibitors FDI-6 and NB-73, or respective control vehicle. The outcomes of colony formation studies revealed that FOXM1i substantially reduced colony numbers in naive and CisR cell lines compared to vehicle treated cells (Fig. 3F–G, supplementary Fig. S1E-F, supplementary Fig. S3A-C). Interestingly, CisR SCLC cell lines exhibited a drastic reduction in colony formation experiments, which is comparable to naïve SCLC cells (Fig. 3G, supplementary Fig. S3A-C). The results of colony formation studies showed an underlying susceptibility of these CR-SCLC cell lines towards FOXM1 inhibition that can be exploited in clinical settings.

SCLC is a highly metastatic disease and 60–70% of SCLC patients are found to have distant metastasis at the time of initial diagnosis [41], which is known as extensive stage SCLC disease [41]. Abrogation of SCLC metastasis is an essential goal for any targeted therapy in SCLC. To study the effect of FOXM1 inhibition on SCLC metastasis in vitro, we performed wound healing and trans-well migration assay. FOXM1 inhibition was able to delay wound healing of CisR H-69 cells (supplementary Fig. S3D). Trans-well migration assays performed on naïve SCLC cells revealed that FOXM1 inhibition with FDI-6 or NB-73 reduced the migratory capabilities of human SCLC cells (supplementary Fig. S4A-B). Trans-well migration assay was also performed on CisR SBC-5 cells, and we observed a consistent significant downregulation in the number of migrated cells following FOXM1i treatment (supplementary Fig. S4C). Similar to the FOXM1 KD cell lines, a spheroid assay was also performed with SBC-5 CisR cells using FOXMi, and we observed consistent and synergistic antitumor response with cisplatin (supplementary Fig. S4D). In conclusion, these results indicate that pharmacological inhibitors of FOXM1 suppress SCLC cell colonization as well as migration capabilities in vitro.

### FOXM1 inhibition induced apoptosis in SCLC cell lines

Following the consistent outcomes from colony formation, cell migration, and reduction in cell viability of SCLC cells, we examined the effect of FOXM1i (FDI-6) on the FOXM1 protein levels. The results of immunoblotting studies showed that FDI-6 treatment depleted FOXM1 levels in SBC-3, SBC-5 and DMS-273 SCLC cell lines (Fig. 3H). To ascertain if FOXM1 inhibition led to apoptosis in SCLC cell lines, we stained the vehicle and FDI-6 treated cell lines with Annexin-V-Cy5 and PI. It was observed that FDI-6 treatment significantly induced apoptosis in multiple SCLC cell lines both from human and mouse, including SBC-3, SBC-5, H-1688, DMS-273, and RPM (Fig. 3I–J, supplementary figure S5). Western blot studies were also performed on multiple SCLC cell lines for accessing cleaved PARP (an apoptotic marker) and it was found that FDI-6 treatment induced cleaved PARP levels in SBC-3, DMS-273 and murine RPM cell lines (Fig. 3K), confirming that FDI-6 induces apoptosis in SCLC cell lines.

### FOXM1 inhibition attenuates SCLC in subcutaneous xenograft mouse model

**Figure 4:**
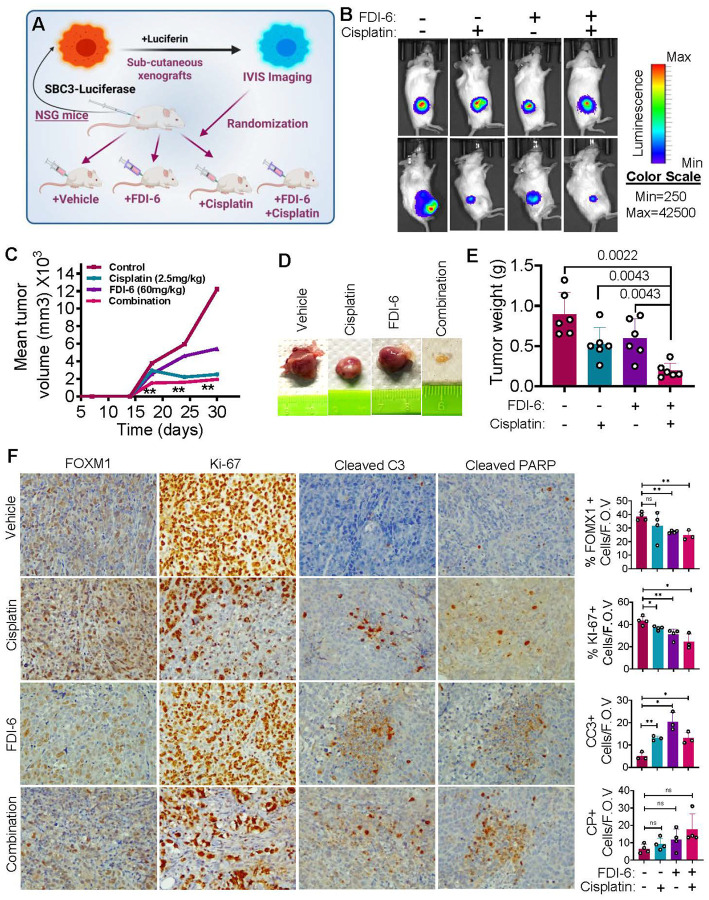
FDI-6 alone or in combination with cisplatin SCLC xenograft tumor growth in vivo. **A)**Representative IVIS imaging of different treatment animals. **B)** Quantification of bioluminescence signal showed downregulation of bioluminescence intensity in treated groups. **C)** Tumor Volume measurement showed decreased tumor growth in the treated animals. **D)** Ex vivo tumors isolated from vehicle, FDI-6 (60 mg/kg), cisplatin (CDDP, 2.5 mg/kg) and combination (FDI-6 and CDDP) groups. **E)** FDI-6 treatment in combination with cisplatin showed significant reduction in tumor growth; tumor weight measured in grams (g). **F)** IHC analysis as indicated for tumor tissues derived from mice treated as in Fig. 4 D. Statistical analysis is from student’s t test, unpaired comparison, ns= non-significant, *=p<0.05, **=p<0.001, and *** = p <0.0001.

To determine the efficacy of FOXM1 inhibition in vivo, we used a human xenograft mouse model in NSG mice, where luciferase labelled SBC-3 cells were subcutaneously injected on the right flank of the mice. Once the mice developed palpable tumors, they were treated with FDI-6 (60 mg/kg), cisplatin (2.5 mg/kg) and their combination (FDI-6 30mg/kg, cisplatin 2.5mg/kg) for 4 weeks (Fig. 4A). IVIS bioluminescence imaging was carried out weekly to follow tumor growth and this imaging showed substantial downregulation of bioluminescence intensity in the different treatment groups (Fig. 4A–B). Tumor measurements were also performed using a slide caliper and volume was calculated. Interestingly, combination treatment utilizing FDI-6 and cisplatin yielded the most significant decrease in bioluminescent intensity and slowed tumor volume progression (Fig. 4A–C). Tumor tissues were harvested, weighed and plotting tumor weight of the different treatment groups showed a significant reduction in tumor weight in the combination therapy group compared to monotherapy groups (Fig. 4D–E). Tissue sections were later processed for immunohistochemical investigations. Prominent downregulation of FOXM1 was observed at tissue level in FDI-6 and combination treated groups (Fig. 4F). However, cisplatin (/CDDP) did not substantially reduce FOXM1 levels in these xenograft tumor tissues. Furthermore, Ki-67 (marker of proliferation) and cleaved caspase 3 (marker of apoptosis) staining showed downregulation of growth and induction of apoptosis in the different treatment groups (Fig. 4F). Together, these data show that FDI-6 in combination with cisplatin attenuates the growth of human SCLC xenografts in vivo.

### FOXM1 inhibition decreases growth of cisplatin-resistant SCLC xenograft tumors

Our in vitro studies suggested that FOXM1i showed efficacies in CisR SCLC cell lines and sensitized them to cisplatin. We further validated the in vitro outcomes using in vivo xenograft models of CisRSCLC cell lines. First, we tested the efficacy of FOXM1i NB-73 on CisR-H69 cell lines and observed a substantial reduction in cell viability following treatment with NB-73 (supplementary Fig. S6A). Later, we immunoblotted FOXM1 after NB-73 treatment and observed that it reduced FOXM1 protein levels and reduced activated ERK signaling (Fig. S6B). Then to study the efficacy of NB-73 in vivo, NSG mice were subcutaneously injected with CisR-H69 cells on the right flank. Once the mice developed palpable tumors, they were randomized and divided into four treatment groups (vehicle group, cisplatin group, NB-73 group and cisplatin and NB-73 combination group). The mice were treated for 4 weeks, and the tumor volume was measured every week with a digital slide caliper. After four weeks of treatment the mice were euthanized, and tumors were harvested alongside different organs. Tumor weight was measured, and it was observed that NB-73 alone or in combination with cisplatin were significantly halting tumor growth of CisR-H69 tumors (supplementary Fig. S6D). The harvested tumors were fixed, and the tumor tissue sections were subjected to IHC staining of cleaved caspase 3 and Ki-67 (proliferation marker). It was found that NB-73 alone as well in combination induces the expression of cleaved caspase 3 and decreased the staining of proliferation marker Ki67 (supplementary Fig. S6E), indicating that NB-73 alone and in combination with cisplatin could effectively decrease the growth of CisR xenograft tumors.

### FOXM1 inhibition downregulates SCLC growth and metastasis in spontaneous mouse model

**Figure 5:**
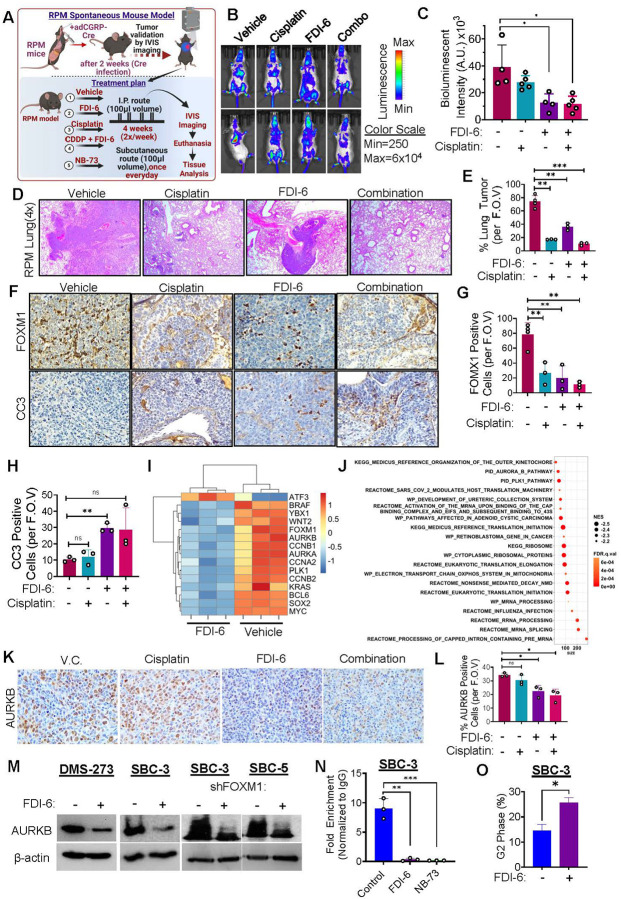
FOXM1 inhibition attenuates growth and metastasis in spontaneous mouse model of SCLC. **A)** Schematic of spontaneous RPM mouse model study. **B)** Representative IVIS Bioluminescence imaging of RPM mice bearing spontaneous lung tumor. **C)** Quantification of bioluminescence imaging intensity in RPM lung in four different treatment groups- vehicle control, FDI-6 (60 mg/kg), cisplatin (CDDP, 2.5 mg/kg) and combination (FDI-6 and cisplatin/CDDP) groups. **D)** H&E Staining of mice Lung showing substantial downregulation of lung tumor size in the combination treatment group compared to control mice. **E)** Quantification of Lung Tumor burden from Figure 5 (D). **F)** IHC staining performed on RPM lung with different treatment groups mentioned in 5 (B) showed FOXM1 and apoptotic marker cleaved caspase 3 (CC3) staining. **G)** Quantification of FOXM1 IHC tissues from Figure 5F. **H)** Quantification of CC3 positive cells shown in Figure 5F. **I)** Bulk mRNA sequencing of DMS-273 showed downregulation of multiple genes after FOXM1 inhibition. **J)** Gene set enrichment analysis showing top upregulated genes in DMS-273 SCLC cell line. **K)** IHC staining performed on SBC-3 xenograft tumor tissues showing positive Aurora Kinase B staining. **L)** Quantification of Aurora Kinase B positive cells shown in Figure 5 (K). **M)** Western blotting analysis depicting downregulation of protein expression followed FOXM1 inhibition and KD. **N)** FOXM1 inhibitors reduces the binding of FOXM1 to Aurora Kinase B target sequence. **O)** FOXM1 inhibition by FDI-6 induces cell cycle arrest in SCLC cells. Statistical analysis is from student’s t test, unpaired comparison, *=p<0.05, **=p<0.001, and *** = p <0.0001.

To further extend our observation closer to human SCLC and investigate a more precise clinical significance of FOXM1i, we evaluated the efficacy of FDI-6 in the RPM spontaneous mouse model, which recapitulates human SCLC characteristics. RPM is a genetically engineered mouse model in which Cre-recombinase knocks out the two major tumor suppressor genes RB1 and P53, and at the same time overexpresses Myc^T58A^-luciferase, a hallmark condition commonly observed in human SCLC tumors [38]. Adenovirus mediated *cgrp*-promoter driven intranasal-cre delivery was used to initiate spontaneous lung tumors in RPM mice (Fig. 5A). Tumor growth was followed by IVIS bioluminescence imaging (Fig. 5B). Once the mice developed a substantial bioluminescence signal, the mice were randomized (Fig. 5A–B) and started on daily treatment with FDI-6 (60 mg/kg), cisplatin (2.5 mg/kg), NB-73 (10mg/kg) and their combination (FDI-6 30mg/kg, cisplatin 2.5mg/kg). Treatment was administered for four weeks and IVIS bioluminescence imaging was performed weekly to follow the progression of spontaneous SCLC tumor and metastasis. Treatment with cisplatin, FDI-6, and combination suppressed the progression of SCLC in RPM mice compared to vehicle and other treatment groups as determined by IVIS bioluminescence intensity (Fig. 5B–C). At the end of the treatment regimen, mice were euthanized, and different organs including lung, liver, brain, kidney, spleen and bones were harvested. Ex vivo imaging showed substantial bioluminescence signal in control lungs compared to other treatment groups (supplementary Fig. S7A). The isolated lung and liver tissues were processed for H&E staining, and it was observed that treatment with cisplatin, FDI-6, and combination (and NB-73) substantially decreased the lung tumor burden with highest effect observed in combination group (Fig. 5D–E, supplementary Figure S7A, S7C). For metastatic burden analysis, we analyzed the liver tissues (one of the primary metastatic sites of SCLC), and it was found that control groups showed large metastatic nodules in the liver, and monotherapy treatment groups showed small metastatic nodules in the liver, whereas no liver metastasis was observed in combination group (supplementary Fig. S7B). Lung tissue sections were further processed for IHC staining for FOXM1, CD31 (a marker of angiogenesis) and cleaved caspase 3 (CC3). IHC staining showed a decrease in FOXM1 levels in FDI-6 and combination treatment groups compared to vehicle group (Fig. 5F–G). Similarly, maximum increase in apoptosis was observed in combination treatment group which was evident by the apoptotic marker CC3 staining (Fig. 5F, 5-H). On the other hand, treatment with cisplatin and FDI-6 alone and in combination resulted in a downregulation of angiogenesis which was measured by CD31 staining (supplementary Fig. S8A). The most prominent downregulation of CD31 was observed in combination treated group (supplementary Fig. S8B-C). These outcomes from spontaneous SCLC mouse model indicates that FOXM1 inhibition using FDI-6 in combination with cisplatin effectively attenuates SCLC progression and metastasis.

### FOXM1 inhibition leads to downregulation of Aurora Kinase B Pathway

To elucidate the mechanism of action of FOXM1 inhibitors, DMS-273 cells were treated with FDI-6 and RNA sequencing experiment was performed. Differential gene expression analysis revealed substantial changes in gene signature. It was observed that many canonical FOXM1 regulated genes like- PLK1, CCNB1, CCNB2 were downregulated (Fig. 5F). Moreover, gene set enrichment analysis revealed that PLK1 and Aurora B pathways were amongst the top downregulated pathways (Fig. 5G). FOXM1 has been reported to regulate Aurora Kinase A and B in previous studies [42]. Recently, the crucial role of FOXM1 has been identified in SCLC [8]. AURKB was known to be a potential therapeutic vulnerability in SCLC [37]; however, FOXM1-AURKB pathway has not been investigated in SCLC. To validate our transcriptomic analysis, immunohistochemical staining was performed for AURKB and pH3, which is a substrate of AURKB, and it was observed that FOXM1i reduced the expression of AURKB and pH3 staining (Fig. 5H, supplementary Fig. S9) in SCLC xenograft tumors. Furthermore, AURKB protein expression was also decreased following pharmacologic inhibition of FOXM1 in SCLC cell lines (Fig. 5I). Following expression studies, a Chip-PCR experiment has been also performed to see if FOXM1 inhibitors impacted FOXM1 binding to the promoter region of AURKB (as well as CDC25B and RRM2) (supplementary Fig. S10A). Interestingly, it was found that FOXM1i (both FDI-6 and NB-73) decreased the direct binding of FOXM1 with AURKB promoter region (Fig. 5J). This shows the effectiveness of FOXM1 inhibitors in downregulating promoter occupancy of FOXM1 and subsequent transactivation of FOXM1 regulated genes.

As FOXM1 and AURKB play a crucial role in cell cycle regulation, we further studied the impact of FOXM1 inhibition on cell-cycle progression of SCLC cells. Interestingly, we observed that FDI-6 treatment halted the cell cycle in G2/M phase in SBC-3 cells (Fig. 5K). Taken together, the mechanistic studies suggest that FOXM1 inhibitors (FDI-6 and NB-73) potently and effectively reduce FOXM1’s promoter occupancy at AURKB target sequence and induce cell cycle arrest. After observing the FOXM1-AURKB relationship in SCLC, specific AURKB inhibitor Barasertib was tested on SBC-3 cells which showed a very potent effect on this cell line, highlighting the importance of FOXM1-AURKB axis (supplementary Fig. S11A). These results indicate that FOXM1-AURKB pathway plays a crucial role in SCLC progression.

### FOXM1 inhibitors potentiate T-cell mediated killing in naïve and CR-SCLC cell line models

**Figure 6:**
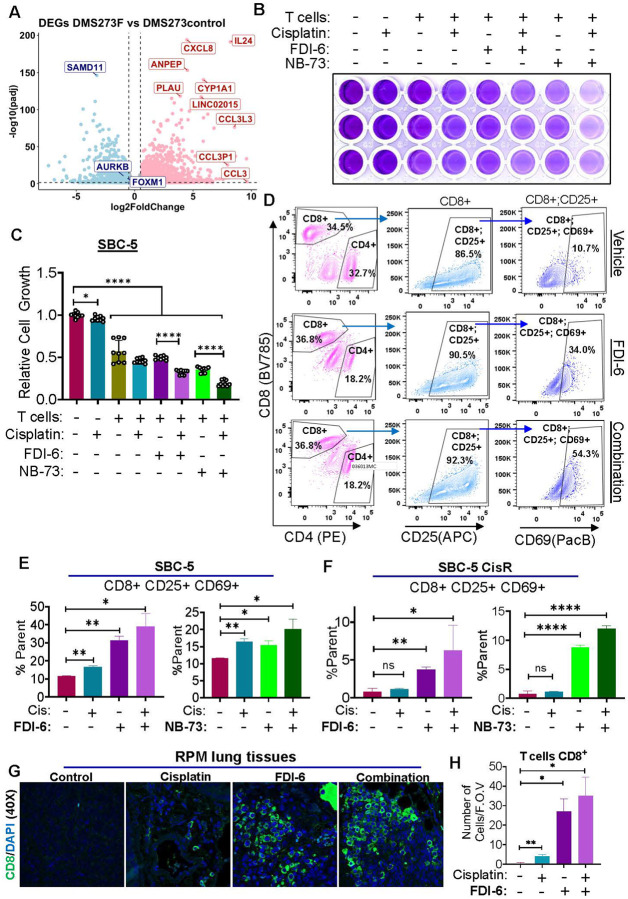
FOXM1 Inhibition in combination with cisplatin potentiates T cell mediated killing of naïve SBC-5 cells. **A)** RNA-sequencing data reveals that FOXM1 inhibition results in the expression of inflammatory cytokines**. B)** FOXM1 inhibitors FDI-6 or NB-73 in combination with cisplatin or alone promotes T cell mediated killing of naïve SCLC cells. **C)** Relative growth rate of SBC-5 cells calculated from the absorbance readings of different treatment conditions. **D)** Representative images of flow cytometry scattering plots used to analyzed T cell activation. **E-F)** Quantitation of human T-cells activation data obtained from co-culture studies of human SCLC cells with T-cells and analyzed through flow cytometry under various treatment conditions. **G)** Representative immunofluorescence staining images from RPM mice lung tumor tissues stained with anti-CD8 antibodies (green) and DAPI (blue) under different treatment conditions. **H)** Quantitation of CD8+ T-cells in RPM lung tumor tissues. student’s t test, unpaired comparison, *=p<0.05, **=p<0.001, *** = p <0.0008, and **** = p< 0.0001.

SCLC tumors are known to create an immunosuppressive microenvironment, and they are typically not inflamed in nature [43, 44]. As a result, these tumor cells modulate the different immune cells, especially T lymphocytes. Moreover, FOXM1 and AURK B have been shown to suppress T cell activity [45, 46]. Further, in our RNA-sequencing data we have observed that FOXM1 inhibition is increasing the expression of inflammatory cytokines (IL24, CXCL8, CCL3, CCL3P1 and CCL3L3) in the SCLC cells which can in turn activate cytotoxic T cells (Fig. 6A). Therefore, we were further interested in investigating the impact of naïve and CisR SCLC cells on human T cell differentiation and activation in the absence and presence of FOXM1i. For this, the human peripheral blood lymphocytes were collected (from UNMC elutriation center) followed by T cell isolation using a negative selection kit as described in methods. Afterwards, the T cells were activated with Dynabeads and co-cultured with cancer cells to study the impact of T cell mediated killing of SCLC cells. Interestingly, it was observed that FOXM1 inhibitors alone or in combination with cisplatin showed synergistic anti-tumor response in naïve and CR-SCLC cell line (Fig. 6B–C, supplementary Fig. S13, S14A). Relative cell growth analysis showed significant potentiation of T cell mediated killing (both in naïve and CR SCLC cell lines) by FOXM1 inhibitors alone and in combination with cisplatin compared to control or vehicle treated cells (Fig. 6C, supplementary Fig. S14B, S15). Another important evidence that we have obtained was expression of immune checkpoint inhibitor PD-L1 in both naïve and CR SCLC cells and augmented levels of PD-L1 were observed in CisR cell lines (supplementary Fig. S16). Expression of PD-L1 explains the immunosuppressive nature of SCLC cells and will have a profound impact on T cell differentiation and activity. To study the impact of SCLC cells on T cell differentiation and activity, a co-culture study was performed followed by multicolor FACS analysis. Flow cytometry analysis revealed a significant increase in activated CD8+ (triple positive T cells: CD8^+^ CD25^+^ CD69^+^) cytotoxic T cells in FOXM1i and cisplatin treatment groups compared to control T cells cocultured with naïve and CR SCLC cells (Fig. 6D–F). This data was validated in immune competent RPM lung tumors, and it was observed that FOXM1 inhibitors alone (FDI-6/NB-73) or in combination with cisplatin (FDI-6) enhanced infiltration of CD8 + cytotoxic T cells in the tumor microenvironment (Fig. 6G–H, supplementary Fig. S20A). We have also observed an increase in expression of immune checkpoint inhibitor PDL-1 after FOXM1 inhibitor treatment in combination with cisplatin (supplementary Fig S20B, S21). The outcomes of T cell activation and differentiation studies suggest that FOXM1 plays a crucial role in the modulation of tumor immune response and the utilization of FOXM1i could be a promising combinatorial approach to enhance the efficacy of immune-checkpoint blockers both in naïve and CR SCLC patients and help to shape the SCLC immune microenvironment towards enhanced antitumor immune response.

## Discussion

Despite the addition of immune checkpoint inhibitors (ICI) and recent approval of Tarlatamab (as a second line therapy), the survival benefits of these therapies in unselected patient population of SCLC is modest and as a result the overall survival of patients is very poor [47]. Targeted therapies are also limited as SCLC is a highly heterogenous disease and most of the known associated modulators (ASCL1, NEUROD1, POU2F3, and PLCG2) are currently undruggable[48]. FOXM1 is a TF that has been recently found to play a critical role in SCLC progression and is associated with poor survival of SCLC patients [9, 15]. However, the detailed mechanism and therapeutic potential of FOXM1 in SCLC remains unclear. We analyzed the FOXM1 expression in publicly available single-cell gene expression data of SCLC patient samples, characterize its expression in multiple cell lines of SCLC (both human and murine), SCLC tissues from genetically engineered mouse model and patient samples, and demonstrated that FOXM1 could be a potential therapeutic target for SCLC. Notably, SCLC is a notorious cancer type that shows high metastasis and relapses with acquired CR, and our study provided the involvement of FOXM1 in the growth, metastasis, modulation of CR and immune response. These outcomes are partly consistent with one recent study in SCLC, where they have shown that FOXM1 plays an important role in SCLC tumorigenesis; however, they didn’t study the role of FOXM1 in metastasis, CR, and modulation of tumor immune cells. Investigating the targeting potential of FOXM1 in SCLC for developing promising therapeutic modalities may help improve survival and quality of life of patients; therefore, we tested FOXM1 inhibitors (FOXM1i) using multiple in vitro and in vivo models of SCLC.

Recent studies reported few specific inhibitors of FOXM1 [31, 33, 34, 49] and in our study, we have chosen two FOXM1 selective inhibitors (FOXM1i), FDI-6 and NB-73 to see their impact on reducing growth and metastasis of SCLC. These small molecules bind with DNA-binding domain of FOXM1 and prevent its interaction with target DNA sequences and show FOXM1 selectivity over other FOXM family members [31, 33]. These inhibitors significantly reduced the viability and cell migratory properties of naïve and CR human as well as murine SCLC cells and induced apoptosis. The emergence of chemotherapeutic resistance (CR) in SCLC is nearly a universal problem, which makes the relapsed tumors difficult to treat. Due to the ineffectiveness of first or second-line therapies, the identification of molecules imparting to CR phenotypes or drugs that can either sensitize the CR cells towards first-line therapy or show synergistic effects with these chemotherapeutic agents are highly desired. To address this challenging issue, we have treated the naïve SCLC cells with low to high doses of cisplatin for ~ 6 months and allow them to phenocopy the conditions of CR. These CR cells showed several-fold (> 10 fold) higher IC_50_ values for cisplatin compared to their naïve counterparts. The protein expression studies on these CR cell lines suggest augmented levels of FOXM1 in CR cells compared to naïve counterparts, suggesting a potential role of FOXM1 in modulating CR of SCLC. Interestingly, the CR-SCLC cells showed high vulnerability towards FOXM1i, which is in accordance with increased FOXM1 expression in CR cell lines compared to respective naïve counterparts (Fig. 2E, supplementary figure S2E-G). Additionally, consistent with 2D culture studies, FOXM1i shows high efficacy in 3D spheroid assays, and SBC-5 CisR cells formed comparatively large spheres then naive SBC-5 cells (Fig. 2G, supplementary Fig. S4D). These 3D spheres are sensitive to FOXM1i, and in combination with cisplatin, FOXM1i sensitizes them to cisplatin, putting forward the involvement of FOXM1 in the modulation of CRSCLC in cells. SCLC cells are known to be extremely metastatic and FOXM1 inhibition significantly reduced cell migration of naïve as well as CR-SCLC cells, and these observation are consistent with the previous study performed in breast cancer cell line, where it was reported that FOXM1i NB-73 decreased the cancer cell migration [33]. FOXM1i alone or in combination with cisplatin showed a substantial reduction in tumor growth in subcutaneous xenograft and spontaneous mouse (RPM) models of SCLC.

Moreover, FDI-6 treated mono/combination therapy treated RPM mice showed significant reduction in the metastatic spread of SCLC at different sites, including liver. Previous studies [8, 9] have not focused on the involvement of FOXM1 in the metastatic potential of SCLC. Moreover, RPM mice are known to generate a heterogenous SCLC tumor [6] with substantial circulating tumor cells and it recapitulates the conditions of human SCLC patients. Substantial reduction of tumor growth and metastasis in RPM model, further strengthens the utilization of FOXM1 as a therapeutic target for developing therapies for SCLC. Since, we did not observe any toxicities, this study advocates further safety tests to be performed that will open doors for a clinical trial in SCLC patients. However, it is interested to note that both the inhibitors of FOXM1 (FDI-6 and NB-73) or genetic knockdown of FOXM1 effectively decreased the cell viability, colonization, and migration of naïve as well as CR-SCLC cell lines, and sensitizes CR cells to cisplatin in vitro and in vivo, providing a strong evidence that these effects are FOXM1 dependent, and it could be a promising molecule to evaluate or target in clinical settings.

AURKB is a viable target in SCLC [6], and our mechanistic studies in SCLC cells treated with FOXM1i coupled with global transcriptomic studies, including Gene Set Enrichment analysis revealed that AURKB is one of the top downregulated pathways, which was further validated in our in vitro and in vivo models. As TFs work through occupying the promoter regions of target genes, we dig-deeper through chromatin immunoprecipitation and demonstrate that FOXM1i reduces occupancy of FOXM1 to target sequence of *AURKB*, and thus, inhibiting its transactivation. FOXM1 and AURKB were known to play a crucial role in mitotic cell division [50]. Because of its role in cell division, inhibiting FOXM1 may adversely affect cell cycle progression in SCLC. AURKB is a part of chromosomal passenger complex (CPC complex), where it serves as the serine/threonine-protein kinase element [51]. CPC complex ensures the proper alignment and segregation of chromosomes, and the complex is also needed for spindle assembly and microtubule stabilization which is induced by chromatin [52–55]. A recent interesting study has been shown that nearly 70–75% of SCLC patients samples have dysregulated cell cycle [56], suggesting that the targeting of FOXM1 would be beneficial to leads to cell cycle arrest in SCLC cells. In addition to FOXM1, our study also provided an alternative way to include AURKB inhibitors to enhance effectiveness or sensitizes CR-SCLC to chemotherapy. Due to the previously established importance of the AURKB pathway in SCLC [37], FOXM1 inhibitors may play a significant role in the treatment of SCLC in the near future.

Therapeutic applications of FOXM1i in SCLC may have supplementary routes of action. For example, we have shown that FOXM1 targeting or FOXM1i modulates the immunosuppressive microenvironment of SCLC. In other cancer types (NSCLC and melanoma), FOXM1 and AURKB have been reported to suppress immune cell activation [44, 45]. Accordingly, the use of FOXM1i might also serve to sensitize SCLC cells or tumors to immune-based therapies, as in our bulk RNA-seq studies following FOXM1i treatment, we have also seen an upregulation of inflammatory chemokines (prominent ones are IL24, CXCL8, and CCL3 and its analogues) gene expression (Fig. S6A), suggesting that FOXM1i may have an impact on T cell activation and T cell-mediated killing of SCLC cells. Indeed, our ex vivo human T-cell killing assays demonstrated an enhanced T cell mediated death in SCLC cells (both in naïve and CR cells) following FOXM1i treatment alone or in combination with cisplatin. Subsequently, T cell activation studies revealed that FOXM1 inhibition increased the T cell activation and supported the differentiation of CD8 + cytotoxic T cells. This data was partially validated by the enhanced infiltration of CD8 + cytotoxic T cells in the tumor immune microenvironment combined with an enhanced expression of immune checkpoint molecules by the spontaneous cancer cells. However, the understanding of precise mechanism of T cell activation is a point of future investigations. One possible mechanism can be the release of inflammatory cytokines by the cancer cells after FOXM1 inhibition as observed in our RNA-seq data. Another possible reason is the association of immune checkpoint molecules with FOXM1. In our studies, we have found that naïve SCLC cells express detectable levels of immune checkpoint molecule PD-L1 and with the increase in FOXM1 levels the expression of PD-L1 further increases (supplementary Fig. S16A). FOXM1 has also been reported to transactivate PD-L1 and therefore, the inhibition of FOXM1 maybe is affecting the ability of SCLC cells to suppress T cell activity [46]. However, the reason behind an increased levels of PD-L1 after FOXM1i treatment still needs further investigation.

## Conclusions

In summary, our study shows that FOXM1 is a potential therapeutic target in SCLC and its inhibition using small molecule inhibitors (FDI-6 or NB-73) elicits effective antitumor response in multiple SCLC cell lines, xenograft, and spontaneous tumor models. FOXM1 levels are substantially high in CR-SCLC cells and FOXM1i in combination with cisplatin is a promising approach to target growth and metastasis of CR-SCLC. This study also provided the mechanistic involvement of FOXM1 and demonstrated that AURKB is a major downstream player of FOXM1 in SCLC. In addition, our study showed that FOXM1i modulate tumor immune cells and enhances immune cell-mediated killing in SCLC cells. There are some limitations to current study, including, FOXM1 low and negative SCLC cell lines and patients will most likely be either non-responders to FOXM1i or will respond insignificantly. In our studies, we have found that the cell lines expressing the high amount of FOXM1, such as CR cell lines, were more susceptible to FOXM1 inhibition compared to their naïve or low FOXM1 expressing counterparts. There is still a gap in understanding the targeting potential of FOXM1 under low vs high conditions. On the other hand, the ability of SCLC cells to generate FOXM1i resistance is still unknown. Therefore, owing to these limitations, the exploration of unidentified roles for FOXM1 such resistance to FOXM1i and/or mechanistic involvement in the modulation of tumor-immune response will provide further exciting opportunities of study.

## Supplementary Material

Supplementary Files

This is a list of supplementary files associated with this preprint. Click to download.

- SupplementaryFiguresS1S21.pdf
- SupplementaryInformation.docx
- SupplementaryFigureLegend1.docx

## Data Availability

The data supporting the results and conclusions of this article are included in this article and its supplementary files. The RNA-Sequencing data generated in this study were deposited to GEO repository with accession number GSE00000 and will be available publicly following the publication of this article.

## Abbreviations

SCLC: Small cell lung cancer
NSCLC: Non-small cell lung cancer
CC3: Cleaved Caspase 3
C. PARP: Cleaved PARP
AURKB: Aurora Kinase B
RNA-seq: RNA-sequencing
PI: Propidium Iodide
FOXM1: Forkhead box protein M1
FOXM1i: FOXM1 inhibitors
